# Accuracies of various types of spinal robot in robot-assisted pedicle screw insertion: a Bayesian network meta-analysis

**DOI:** 10.1186/s13018-023-03714-8

**Published:** 2023-03-25

**Authors:** Lin-Zhen Xie, Qi-Long Wang, Qi Zhang, Da He, Wei Tian

**Affiliations:** 1 Department of Spine Surgery, Peking University Fourth School of Clinical Medicine, Beijing, China; 2 Department of Spine Surgery, Beijing Jishuitan Hospital, Beijing, China; 3 Research Unit of Intelligent Orthopedics, Chinese Academy of Medical Sciences, Beijing, China

**Keywords:** Pedicle screw, Robot, Robot‐assisted, Spine, Network meta-analysis

## Abstract

**Background:**

With the popularization of robot-assisted spinal surgeries, it is still uncertain whether robots with different designs could lead to different results in the accuracy of pedicle screw placement. This study aimed to compare the pedicle screw inserting accuracies among the spinal surgeries assisted by various types of robot and estimate the rank probability of each robot-assisted operative technique involved.

**Methods:**

The electronic literature database of PubMed, Web of Science, EMBASE, CNKI, WANFANG and the Cochrane Library was searched in November 2021. The primary outcome was the Gertzbein–Robbins classification of pedicle screws inserted with various operative techniques. After the data extraction and direct meta-analysis process, a network model was established in the Bayesian framework and further analyses were carried out.

**Results:**

Among all the 15 eligible RCTs, 4 types of robot device, namely Orthbot, Renaissance, SpineAssist and TiRobot, were included in this study. In the network meta-analysis, the Orthbot group (RR 0.27, 95% CI 0.13–0.58), the Renaissance group (RR 0.33, 95% CI 0.14–0.86), the SpineAssist group (RR 0.14, 95% CI 0.06–0.34) and the conventional surgery group (RR 0.21, 95% CI 0.13–0.31) were inferior to the TiRobot group in the proportion of grade A pedicle screws. Moreover, the results of rank probabilities revealed that in terms of accuracy, the highest-ranked robot was TiRobot, followed by Renaissance and Orthbot.

**Conclusions:**

In general, current RCT evidence indicates that TiRobot has an advantage in the accuracy of the pedicle screw placement, while there is no significant difference among the Orthbot-assisted technique, the Renaissance-assisted technique, the conventional freehand technique, and the SpineAssist-assisted technique in accuracy.

**Supplementary Information:**

The online version contains supplementary material available at 10.1186/s13018-023-03714-8.

## Introduction

The precise insertion of pedicle screws plays a central role in pedicle screw fixation, which provides three-column stabilization and is widely used in spine surgery. There were considerable variabilities in the anatomy of vertebral landmarks and the structure of pedicles due to specific physical or pathological variations. Even if pedicle screw insertion is the most foundational operation for spine surgeons, it is still hard to insert all pedicle screws precisely by the conventional freehand method [1]. Deviations in the placement without deviation are associated with a reduction of biomechanical strength and even a series of complications such as spinal instability, vascular injuries, and neurologic injuries [2].

With the advance of computer-assisted navigation technology and spinal robot technology, traditional pedicle screw placement has been partially replaced. Both navigation technology and robotic technology guide screw placement according to the three-dimensional mapping of the vertebrae; the major difference being surgeon-guided vs. robot-guided execution of the screw trajectory [3, 4]. There is no denying that the arrival of the spinal operative robot is a potential milestone in the development of spine surgery. With the maturity of robot technology, some revolutionary spinal robot products have been applied in clinical practice. Most spinal robot products are originally designed for navigation and positioning during operation. Therefore, these robots are especially suitable for the placement of pedicle screws. The application of spinal robots shows excellent promise in avoiding the drawbacks of the conventional freehand method, such as the instability of human hands. Consequently, robot-assisted spinal surgery is becoming increasingly prevalent.

In the comparison of robot-assisted surgery and conventional spinal surgery, the accuracy of pedicle screw insertion seems controversial. Even though most previous studies support the conclusion that the use of spinal robots can increase accuracy in pedicle screw insertion [5, 6], there are also some contradictory arguments [7, 8]. Moreover, there is no direct comparison report or intensive study on different types of spinal robot. It is still uncertain whether robots with different designs and structures can lead to different clinical results, such as the accuracy of pedicle screw placement.

The Bayesian network meta-analysis (NMA) approach allows for comparing methods that have never been compared directly through the common comparator. Therefore, the purpose of this NMA was to indirectly compare the pedicle screw inserting accuracies among the spinal surgeries assisted by various types of robot by evaluating data from published prospective randomized controlled trials. Furthermore, the rank probability of each particular spinal robot was estimated based on the Bayesian approach.

## Materials and methods

This Bayesian network meta-analysis was reported according to the PRISMA for Network Meta-Analyses (PRISMA-NMA) statement [9]. Moreover, this research was registered in the PROSPERO database (CRD42021288938).

### Searching and screening strategy

Up to November 2021, a comprehensive search was performed through PubMed, Web of Science, EMBASE, CNKI, WANFANG, and the Cochrane Library using a combination of the terms “robot-assisted,” “robotic,” “robot-aided,” “spine,” ”spinal” and “pedicle screw.” Two independent authors carried out the searching process without language limitation and screened records by title and abstracts. Then, probably eligible reports were examined with the same eligibility criteria. Discrepancies were discussed in the group meeting all members reached an agreement.

### Eligibility criteria

Inclusion criteria were as follows: (a) prospective randomized controlled trials; (b) clinical trials performed on human beings; (c) adult patients (older than 18 years old); (d) patients with degenerative or traumatic spinal diseases; (e) patients undergoing lumbar, thoracolumbar or lumbosacral spine surgery with pedicle screw implantation; (f) comparison of the robot-assisted operative method and conventional operative method or two robot-assisted operative methods; (g) Accuracy of pedicle screw placement was evaluated by Gertzbein–Robbins Scale. Exclusion criteria were as follows: (a) cadaveric studies; (b) patients with spinal scoliosis; and (c) conference articles, editorials, or letters.

### Quality assessment and data extraction

For each eligible report, two independent authors evaluated the quality according to the Cochrane risk of bias assessment scale, and the data were then extracted independently. The extracted items were as follows: (a) authors; (b) year of publication; (c) country; (d) study design; (e) disease; (f) the type of surgery; (g) robot devices; (h) the age of patients; (i) the gender of patients; (j) the number of patients; (k) instrumented vertebral body; (l) the number of pedicle screws; (m) pedicle screws classified by Gertzbein–Robbins Scale, etc. During the assessing and extracting process, the whole team checked and discussed all discrepancies.

### Data analysis

The meta-analysis and Network meta-analysis were conducted using STATA statistical software and ADDIS software. *P* < 0.05 was set as the level of significance. The Risk ratio (RR) and corresponding 95% confidence interval (CI) were used to compare dichotomous variables. The random-effects model was used regardless of the heterogeneity. Moreover, the statistical heterogeneity was assessed with the forest plot and inconsistency statistic (I-squared). The funnel plot was used to evaluate reports for publication bias. As for the Bayesian network analysis, a network consistency model was built with Markov chain Monte Carlo methods [10]. The translated binary results of pedicle screw placement were networked and the relations among the risk ratios across the network were specified, as Cipriani et al. reported [11]. The RR of each operative procedure was calculated, and the proportion of iterations of the Markov chain of the RR ranking was counted so that the rank probability of each operative procedure could be estimated. The cumulative ranking curve and surface under the cumulative ranking curve (SUCRA) were also conducted. *P* < 0.05 and 95% CIs beyond the null value were adopted to assess the significance.

## Results

### Studies involved

**Fig. 1:**
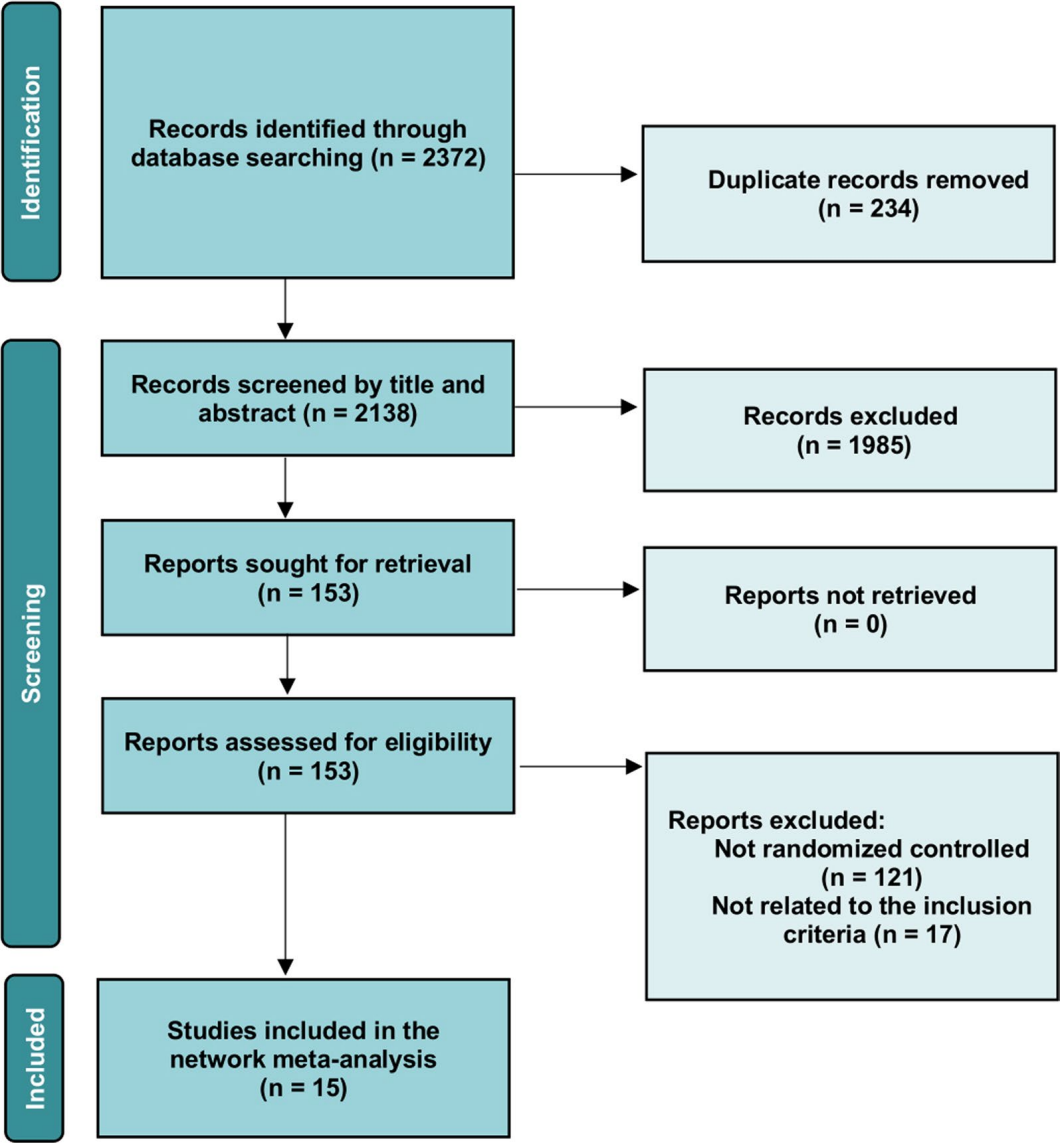
The flowchart of the searching and screening process

**Fig. 2:**
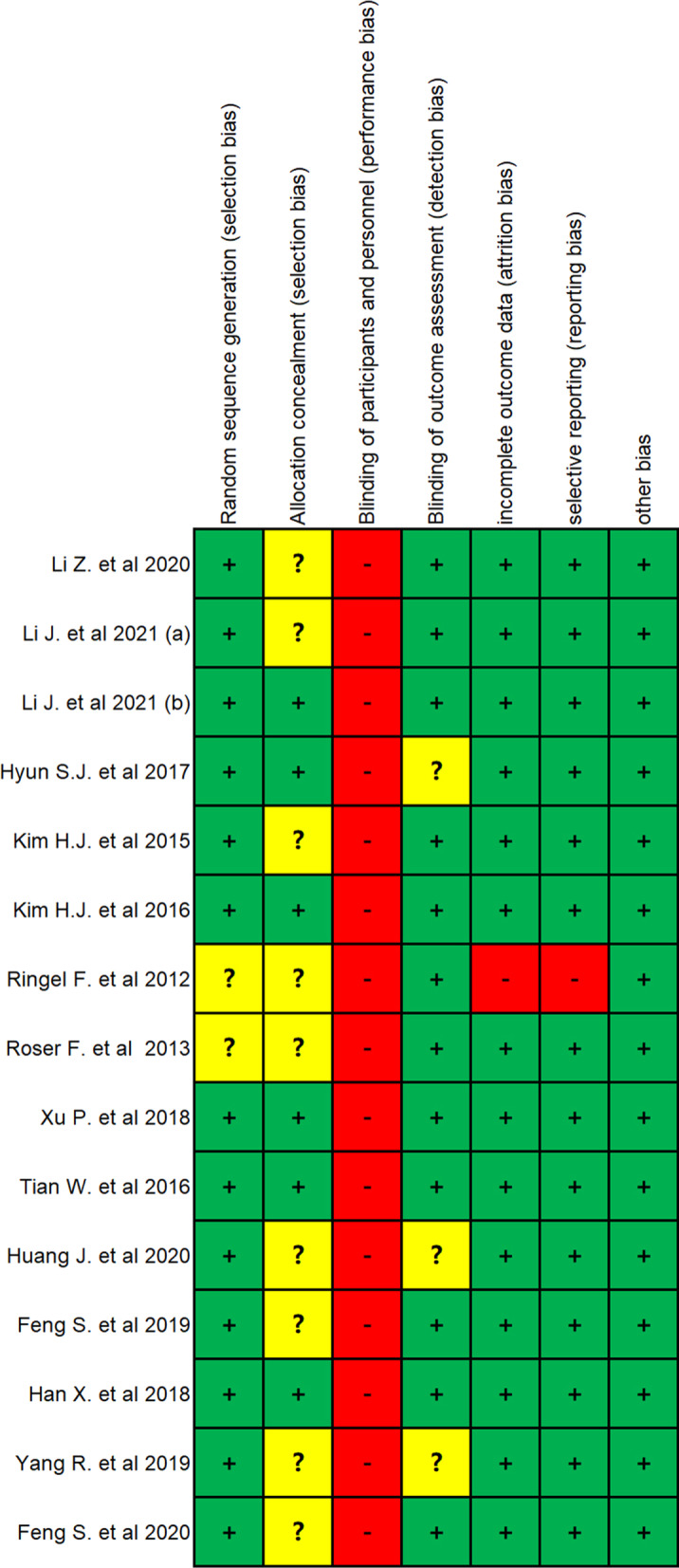
Risk of bias summary: green for low risk of bias, yellow for unclear bias and red for high risk of bias

**Fig. 3:**
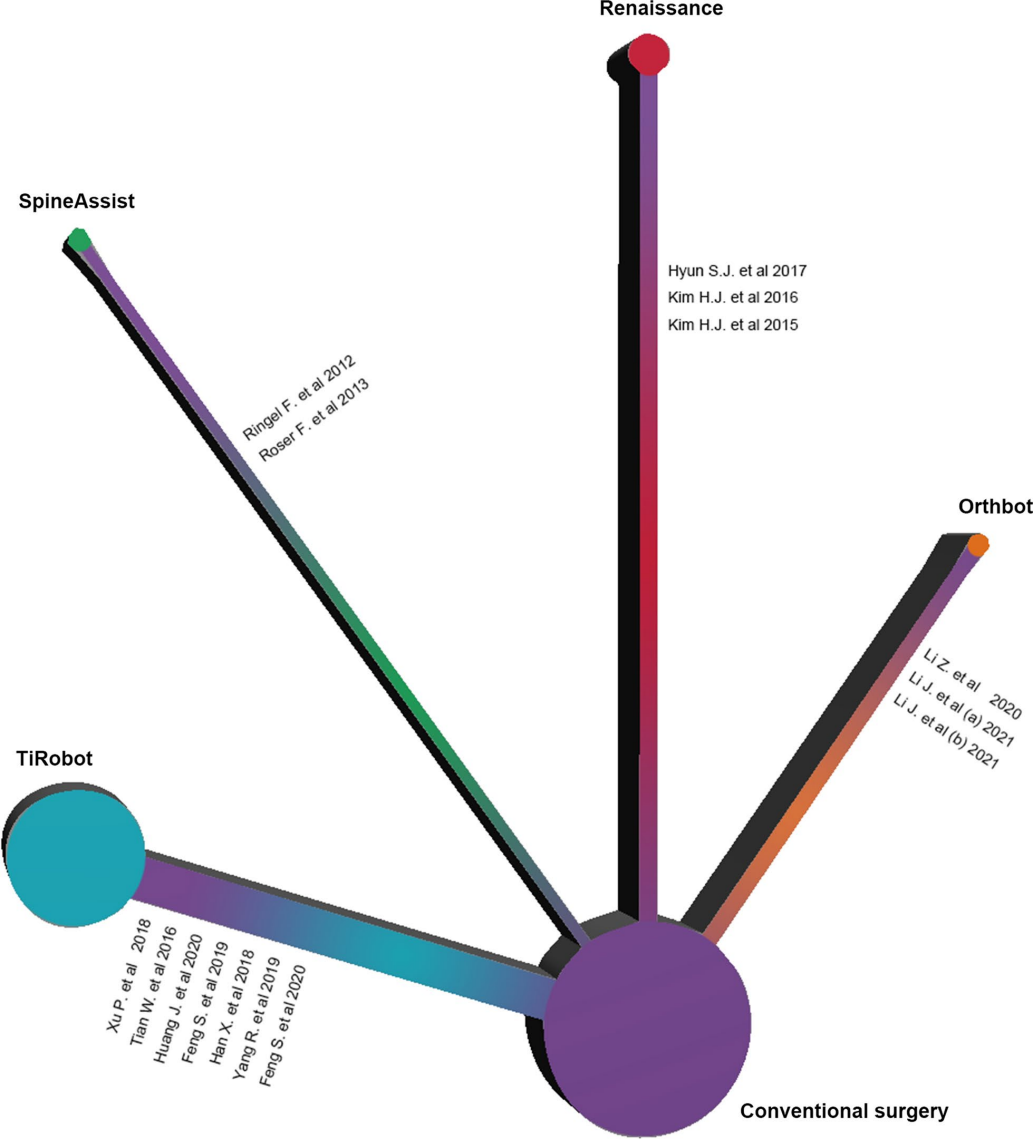
The network plot of spinal operation with freehand and different robot types. The size of the node represents the number of patients. The line between nodes represents the existence of direct comparison, and the width of lines is proportional to the number of studies

A total of 2372 candidate records were identified through database searching. After removing duplicates, 2138 records were screened by title and abstract. Of the 153 potential studies further assessed with the full text, 138 were excluded according to the inclusion and exclusion criteria details. The final number of eligible studies involved was fifteen (Fig. 1). The results of the quality assessment are shown in Fig. 2. The characteristics of the eligible studies are summarized in Table 1. Overall, 988 patients and 4594 pedicle screws were included in this study. There is no direct comparative study on different types of spinal robot device. All the studies were randomized controlled trials involving the comparison of the conventional spinal operation and the robot-assist operation with one particular type of spinal robot. Four types of robot device, namely Orthbot [12–14], Renaissance [8, 15, 16], SpineAssist [7, 17] and TiRobot [18–24], were included (Fig. 3). All 15 studies used the Gertzbein–Robbins Scale to describe early radiological outcomes of pedicle screws.

**Table 1:** The summary table of the information extracted from eligible studies

Author	Year	Country	Robot device	Disease	Instrumented vertebral body	Age	Male/female	Study design	Number of patients underwent conventional surgery	Number of pedicle screws under conventional method						Number of patients underwent robot-assisted surgery	Number of pedicle screws under robot-assisted method					
										Overall	Grade A	Grade B	Grade C	Grade D	Grade E		Overall	Grade A	Grade B	Grade C	Grade D	Grade E
Li et al.	2020	China	Orthbot	Degenerative lumbar disease or lumbar spinal stenosis	Lumbar or lumbosacral	23 ~ 63	7/10	RCT	10	50	39	10	1	0	0	7	32	29	3	0	0	0
Li et al. (a)	2021	China	Orthbot	Lumbar spinal stenosis	Lumbar or lumbosacral	40 ~ 60	29/27	RCT	29	136	98	38	0	0	0	27	128	94	34	0	0	0
Li et al. (b)	2021	China	Orthbot	Lumbar spinal stenosis	Lumbar or lumbosacral	40 ~ 60	10/14	RCT	11	48	32	16	0	0	0	13	58	42	16	0	0	0
Hyun et al.	2017	South Korea	Renaissance	Degenerative lumbar disease	Lumbar or lumbosacral	67 (mean)	17/43	RCT	30	140	133	5	1	1	0	30	130	127	3	0	0	0
Kim. et al.	2016	South Korea	RenaissanceL	Lumbar spinal stenosis	Lumbar or lumbosacral	40 ~ 80	41/37	RCT	41	172	158	13	1	0	0	37	158	148	9	1	0	0
Kim et al.	2015	South Korea	Renaissance	Degenerative lumbar disease	Lumbar or lumbosacral	40 ~ 85	19/21	RCT	20	80	73	6	1	0	0	20	80	76	4	0	0	0
Ringel et al.	2012	German	SpineAssist	Lumbar instability	Lumbar or lumbosacral	67 (median)	26/34	RCT	30	152	103	38	5	5	1	30	146	82	42	16	5	1
Roser et al.	2013	German	SpineAssist	Vertebral body fracture or degenerative instability	Lumbar or lumbosacral	18 ~	NA	RCT	10	40	39	1	0	0	0	18	72	71	0	1	0	0
Xu et al.	2018	China	TiRobot	Thoracolumbar fracture	Thoracolumbar	47 (mean)	25/18	RCT	19	106	78	15	9	4	0	24	132	127	5	0	0	0
Tian et al.	2016	China	TiRobot	Lumbar fracture or lumbar spondylolisthesis	Lumbar or lumbosacral	54 (mean)	17/23	RCT	17	95	88	6	1	0	0	23	102	99	3	0	0	0
Huang et al.	2020	China	TiRobot	Degenerative lumbar disease	Lumbar or lumbosacral	55 (mean)	33/27	RCT	32	128	108	12	7	1	0	28	112	106	5	1	0	0
Feng et al.	2019	China	TiRobot	Degenerative lumbar disease	Lumbar or lumbosacral	68 (mean)	25/55	RCT	40	225	206	18	1	0	0	40	202	199	3	0	0	0
Han et al.	2018	China	TiRobot	Thoracolumbar degenerative or traumatic disorders	Thoracolumbar	18 ~ 80	113/121	RCT	119	584	503	43	27	8	3	115	532	507	18	5	2	0
Yang et al.	2019	China	TiRobot	Thoracolumbar fracture or degenerative lumbar disease	Thoracolumbar, lumbar or lumbosacral	45 (mean)	49/39	RCT	43	202	164	22	15	1	0	45	208	203	5	0	0	0
Feng et al.	2020	China	TiRobot	Degenerative lumbar disease	Lumbar or lumbosacral	64 (mean)	31/49	RCT	40	174	162	11	1	0	0	40	170	167	3	0	0	0

*NA* not applicable; *RCT* randomized controlled trial

### Direct meta-analysis

**Fig. 4:**
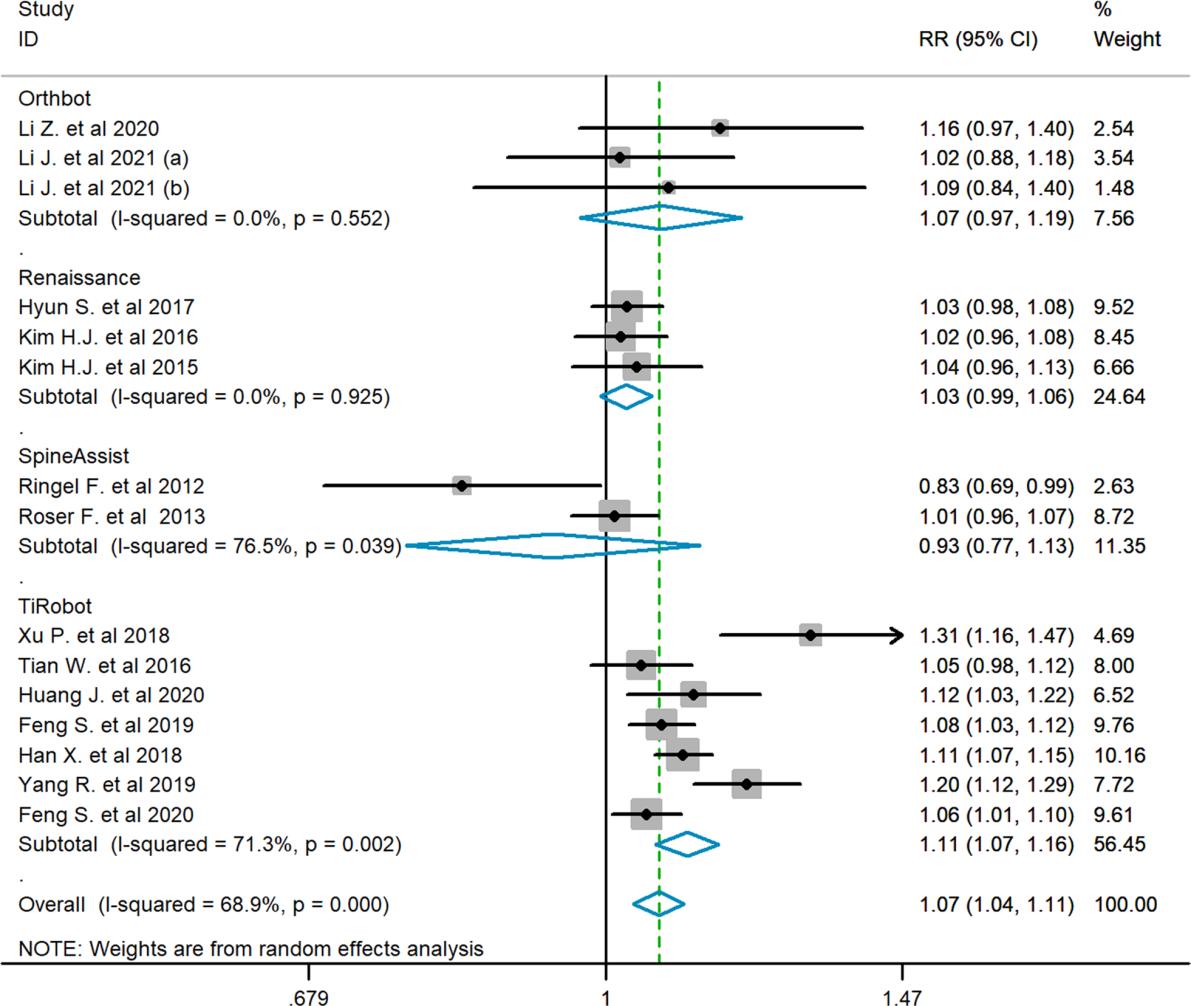
Forest plot showing the direct meta-analysis of the Gertzbein–Robbins classification of pedicle screws inserted with various operative techniques

Based on the Gertzbein–Robbins classification outcome reported by eligible studies, the direct meta-analysis revealed a significant difference between the overall robot-assisted operation group and the conventional group in the proportion of grade A pedicle screws (RR = 1.07, 95% CI 1.04–1.11). Further subgroup analysis by the type of robot device was carried out due to the existence of heterogeneity (I-squared = 68.9%, *p* < 0.001). In terms of the comparison to the conventional group in the proportion of grade A pedicle screws, the TiRobot subgroup (RR = 1.11, 95% CI 1.07–1.16, I-squared = 71.3%, *p* = 0.002) was detected with a significant difference, while the subgroups of Orthbot (RR = 1.07, 95% CI 0.97–1.19, I-squared = 0.0%, *p* = 0.552), Renaissance (RR = 1.03, 95% CI 0.99–1.06, I-squared = 0.0%, *p* = 0.925) and SpineAssist (RR = 0.93, 95% CI 0.77–1.13, I-squared = 76.5%, *p* = 0.039) were not significant (Fig. 4).

### Network meta-analysis

**Fig. 5:**
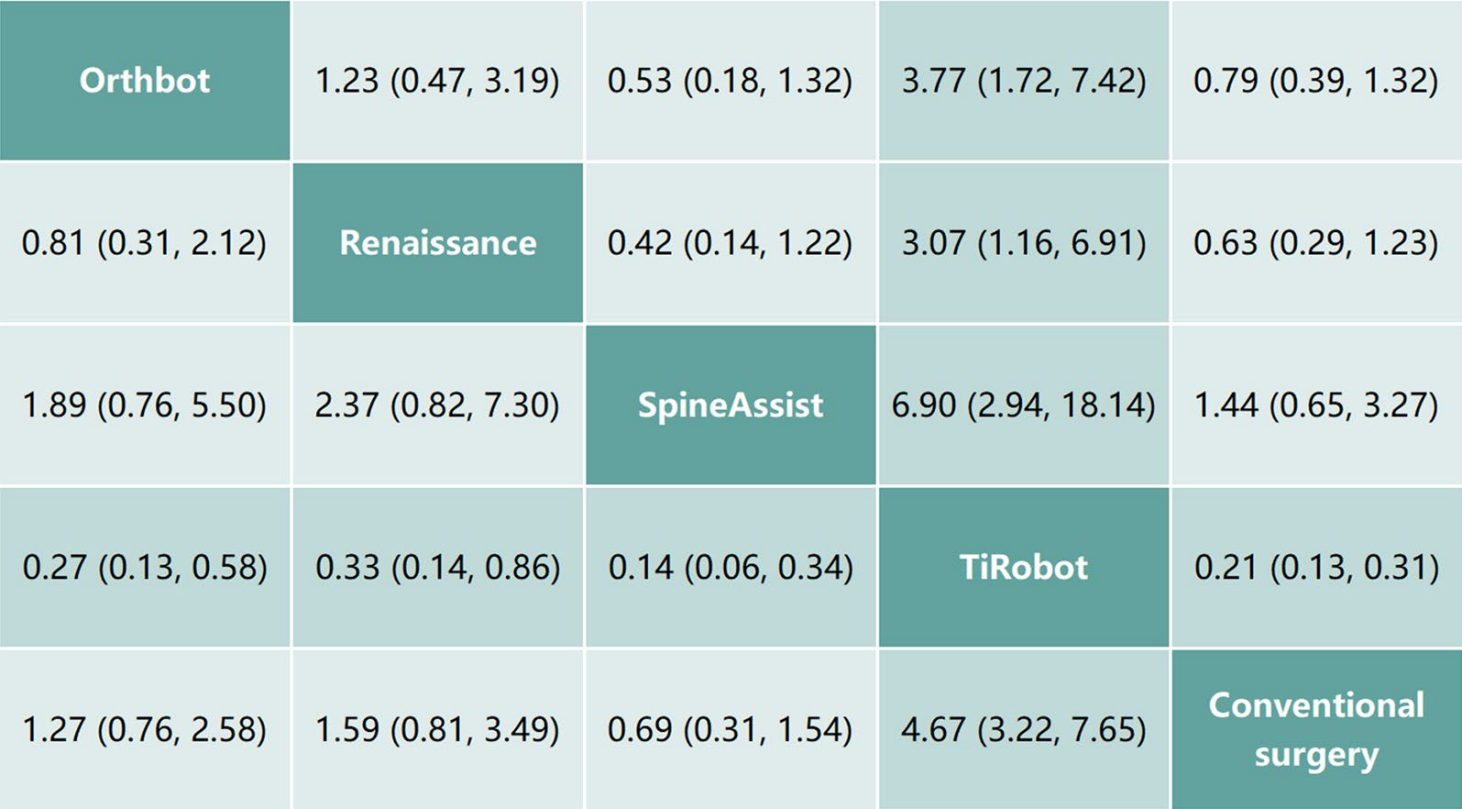
Multiple comparisons for pedicle screw inserting accuracy based on the network consistency model. Results are presented as relative risks with 95% confidence intervals

In light of the Bayesian approach, a consistency network model was established from the studies involved, and the median of variance parameters of which was 0.23 (95% CrI 0.01–0.77). According to the proportion of pedicle screws without breach of the cortical layer of the vertebral body or pedicle (Gertzbein–Robbins grade A), Fig. 5 summarizes the league tables with RR and 95% CIs of operative methods compared with each other. Taking the TiRobot group as reference, the Orthbot group (RR 0.27, 95% CI 0.13–0.58), the Renaissance group (RR 0.33, 95% CI 0.14–0.86), the SpineAssist group (RR 0.14, 95% CI 0.06–0.34) and the conventional surgery group (RR 0.21, 95% CI 0.13–0.31) showed significantly lower rates of grade A screws.

### Rank probabilities

**Fig. 6:**
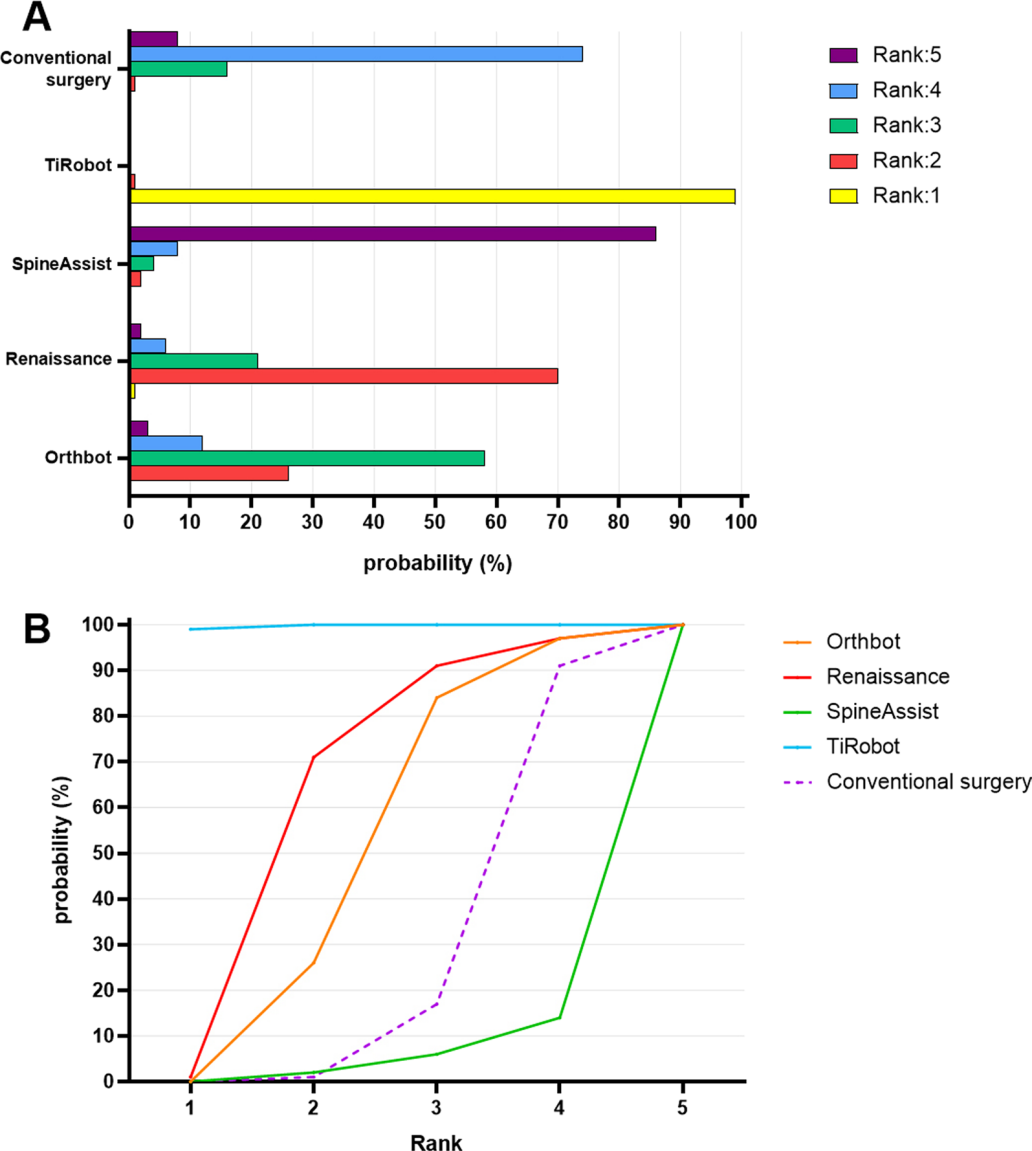
**A** Histogram showing the distribution of ranking probabilities of each operative technique in accuracy. **B** Cumulative ranking plots comparing each of the operative techniques in pedicle screw inserting accuracy

On the basis of the network model, the rank probability of each operative method was estimated (Fig. 6A). Each color represented one accuracy hierarchy, and the overall distribution was represented by histogram. Similarly, Fig. 6B shows the cumulative probabilities for each operative method at each possible rank. Larger SUCRA indicated better rank among all involved operative methods. Specifically, the SUCRA of the Orthbot group, the Renaissance group, the SpineAssist group, the TiRobot group and the conventional surgery group were, respectively, 64.0%, 77.8%, 18.0%, 99.8% and 40.0%.

## Discussion

In the wake of developments in science and technology, the dream of robotic spinal surgery has come into reality. Even though most current spinal robot products were mainly designed for navigation and positioning during operation, these revolutionary robot devices can provide tremendous assistance in spinal surgery. Although designed by different companies and endowed with particular characteristics, these spinal robots have some similarities in both structure and function. And the vast majority of these robots, including SpineAssist (2004, Mazor Robotics Ltd., Caesarea, Israel), Renaissance (2011, Mazor Robotics Ltd., Caesarea, Israel), Excelsius GPS (2017, Globus Medical, Inc., Audubon, Pennsylvania, USA), TiRobot (2016, TINAVI Medical Technologies, Beijing, China), Orthbot (2019, Xin Junte, Shenzhen, China), and Mazor X Stealth Edition (2019, Medtronic, Memphis, TN, USA), are shared-control robots which allow both the robot and operator to control motions simultaneously [25, 26].

SpineAssist, the first shared-control robot approved by the FDA for use in spine surgery, mounts onto vertebral landmarks directly and aligns its arm along the designed trajectory. The location of the entry point and the direction for the guide wire are thus determined [27]. SpineAssist can merge preoperative CT with intraoperative fluoroscopy, and the planning of the target trajectory relies on CT imaging. Renaissance, the successor of SpineAssist, also requires a patient-mounted track. Renaissance contains some improvements in hardware and software on the basis of the first-generation Mazor robot, such as improved artifact rejection, faster image processing speed, and the ability to flatten bone at the entry points in order to avoid skiving [28, 29]. Neither SpineAssist nor Renaissance is equipped with integrated navigation [28]. Computer-assisted navigation allows for clearer visualizations of structural anatomy during spinal surgery [30]. After years of development, navigation technology has evolved from early two-dimensional navigation based on fluoroscopy to intraoperative computed tomography-based navigation [31]. The development of both computer-assisted navigation and spinal robot technology complements each other; in fact, many spinal robots are even considered special applications of three-dimensional navigation [32]. Most of recent spinal robot productions have been integrated with navigation systems. Excelsius GPS addresses several shortages of many previous spine robots, such as inaccurate registration and inaccurate navigation. The tubular robotic arm is able to withstand significant lateral force without displacement. Therefore, Excelsius GPS allows for screw placement without the usage of Kirschner wires [33]. TiRobot, a multi-indication orthopedic robot, is consisted of an optical tracking device, a robotic arm and a robotic workstation. The movement of the robotic arm is under the supervision of the stereo-tracking device. The intraoperative 3D navigation image, acquired from the C‐arm scanner and reconstructed by the workstation, can help to carry out the planning of the screw trajectories by checking trajectories and anatomical landmarks in real time. Before inserting the screw, a Kirschner wire has to be drilled along the robotic guided cannula as the guiding pin [26]. Though most spine robots are incapable of automatic drilling, Orthbot is an exception. A bone drill is combined into the mechanical arm, which is composed of a motor and a force sensor that can monitor the drilling pressure. Therefore, Orthbot has the capability to drill and insert the Kirschner wire autonomously [14]. As the latest generation of Mazor spine robot, Mazor X Stealth Edition includes an intraoperative O-arm scan, which makes it possible to provide real-time visualization of the drilling and screw placement process. In addition, Mazor X Stealth Edition obviates the need for a percutaneous pin and the need for a preoperative CT scan [34]. Being considered as representatives, these robots signify the inheritance and developmental direction for spinal robots in some ways; nonetheless, previous clinical studies have not yet provided enough high-quality evidence for each type of robot. According to the current result of the literature search, SpineAssist, Renaissance, TiRobot, and Orthbot have been investigated by randomized controlled trials. Therefore, these four types of spinal robot were the research objects of this study.

**Fig. 7:**
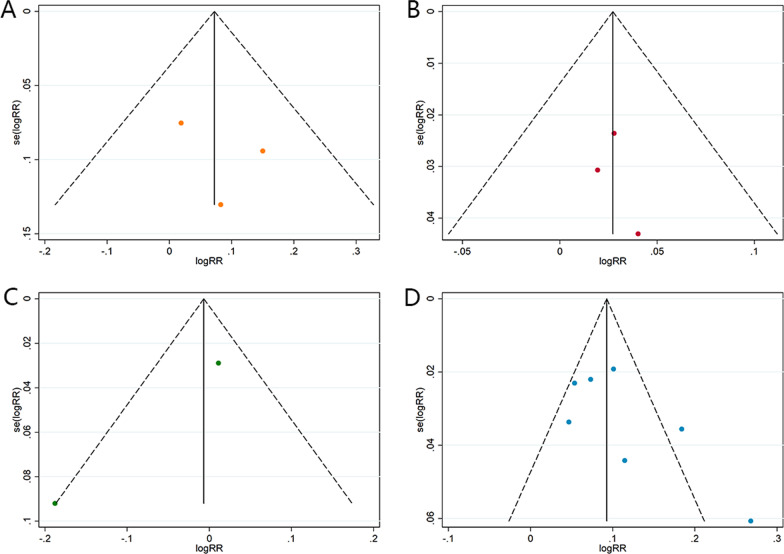
**A** The funnel plot of the Orthbot group. **B** The funnel plot of the Renaissance group. **C** The funnel plot of the SpineAssist group. **D** The funnel plot of the TiRobot group

In accordance with previous reports [5, 6, 35], the result of the direct meta-analysis ((RR = 1.07, 95% CI 1.04–1.11)) confirms that the robot-assisted method is superior to the conventional technique in accuracy. Moreover, further subgroup analysis by the type of robot device shows the main positive effect is original from the subgroup of TiRobot (RR = 1.11, 95% CI 1.07–1.16). Moreover, no obvious publication bias is found in the funnel plot (Fig. 7). However, almost all previous research and traditional meta-analyses focused on the difference between the freehand technique and robot-assisted technique in spine surgery. In terms of various types of spinal robot, there was not sufficient research or even direct literature evidence.

Through the common comparator, the NMA approach could compare various operative techniques indirectly without direct evidence. In order to compare the accuracies of different types of spinal robot, the Bayesian framework network was established so that the indirect connection of robot-assisted techniques could be linked through the common comparator technique, and indirect evidence could be further incorporated. The NMA has been recognized as a credible approach to comparing various treatments or techniques for its high consistency with reality [36]. The TiRobot-assisted technique shows higher accuracy than other techniques, while no significant difference was detected among other techniques. In addition, the Bayesian chain can be used to quantify the uncertainty and rank these techniques by measuring the corresponding probability [37]. In terms of the accuracy of pedicle screw placement, the TiRobot-assisted technique has a great probability of ranking first, followed by the Renaissance-assisted technique, the Orthbot-assisted technique, the conventional freehand technique and the SpineAssist-assisted technique.

The four types of spinal robot involved in this study, namely SpineAssist (entering the market in 2004), Renaissance (entering the market in 2011), TiRobot (entering the market in 2016) and Orthbot (entering the market in 2019), represent the inheritance and development history of robot-assisted spinal surgery to some extent. Compared with SpineAssist, the second-generation counterpart Renaissance has some improvements in both software and hardware. But it is reported that these two robots face a similar problem of screw misplacement secondary to skiving [27, 38], which may partially explain the rank probabilities (Fig. 6). With the progress of technology, the automation and intelligence levels of spinal robots have increased gradually. Orthbot is even capable of drilling and inserting the Kirschner wire autonomously. However, the introduction of the automatic drilling function might be associated with some losses in accuracy. The accuracy of the screw placement assisted by Orthbot is not significantly different from the conventional freehand group or the Renaissance group but inferior to the TiRobot group. Generally, the automation and accuracy of spinal robots gradually increase in the process of inheritance and development. According to the surface under the cumulative ranking curve, TiRobot ranks first on the accuracy of pedicle screw placement, followed by Renaissance, Orthbot, conventional technique, and SpineAssist. Launched in 2016, TiRobot reaches a peak in terms of accuracy. Along with the introduction of the automatic drilling function, the more recent Orthbot operates with a potential accuracy loss, while there is no significant difference among the Orthbot-assisted technique, the Renaissance-assisted technique, the SpineAssist technique, and the conventional technique in pedicle screw inserting accuracy based on the Bayesian network.

In this study, there were quite large differences in the number of studies and sample sizes between groups. The design of the Bayesian method represents dependencies between multiple variables as conditional probability distributions [39]. Accordingly, we could explicitly eliminate these differences by quantifying the joint probability of each group, obtaining objective comparison results. For the TiRobot group with the largest sample size, there was significant heterogeneity within the group (I-Squared = 71.3%). As a consequence, we conducted further influence analysis on the TiRobot group (Additional file 1: Fig. S1). Combining the results of the influence analysis and forest plot (Fig. 4), the study by Xu et al. [18] was the largest outlier effect source, which may have overestimated the positive effects of TiRobot. After excluding this study, the heterogeneity of the TiRobot group was significantly reduced (I-Squared = 59.4%), and the result of the direct meta-analysis remained significant (Additional file 1: Fig. S2). Compared to the original analysis, the resulting consistency network model of Bayesian network meta-analysis achieved a similar median variance parameter of 0.21 (95% CrI 0.01–0.75). Although the results changed slightly in numerical terms, the final conclusion remained unchanged (Additional file 1: Figs. S3 and S4). In addition, due to the limitation of the number of studies, other groups could not be further analyzed this way.

There are several limitations of this Bayesian network meta-analysis. Firstly, even if we have tried to select studies with relative homogeneity and high quality of evidence by setting strict criteria, some heterogeneities exist among the studies involved. Though clinical heterogeneity is inevitable, it makes little impact on the final conclusion. Secondly, accuracy is undoubtedly the most concerning outcome for robot-assisted spinal surgery, while other indexes such as radiation exposure, operative time, bleeding amount, and complication rate are also important. But currently available evidence cannot support comprehensive network meta-analyses for outcomes except for the accuracy of screw placement.

## Conclusions

Current RCT evidence indicates that TiRobot has an advantage in the accuracy of the pedicle screw placement, while there is no significant difference among the Orthbot-assisted technique, the Renaissance-assisted technique, the conventional freehand technique, and the SpineAssist-assisted technique in accuracy. With the popularization of robot-assisted spinal surgeries and the emergence of new spinal robots, more well-designed randomized controlled trials and further studies are required in future.

## Supplementary Information

**Additional file 1**. Further analyses related to the heterogeneity of the TiRobot group.

## Data Availability

The data are available from the corresponding author on reasonable request.

## Abbreviations

RR: Relative risk
CI: Confidence interval
RCT: Randomized controlled trials
PRISMA: Preferred reporting items for systematic reviews and meta-analyses
NMA: Network meta-analysis
CrI: Credible interval
FDA: Food and Drug Administration
SUCRA: The surface under the cumulative ranking curve
