# Supplementary material for: Accuracies of various types of spinal robot in robot-assisted pedicle screw insertion: a Bayesian network meta-analysis
Source: J Orthop Surg Res. 2023 Mar 25;18:243. doi: 10.1186/s13018-023-03714-8 (PMC10039560; doi:10.1186/s13018-023-03714-8)
Supplement: Supplementary file 1 — Additional file 1. Further analyses related to the heterogeneity of the TiRobot group. [file 13018_2023_3714_MOESM1_ESM.docx]

**Additional file 1: Further analyses related to the heterogeneity of the TiRobot group**


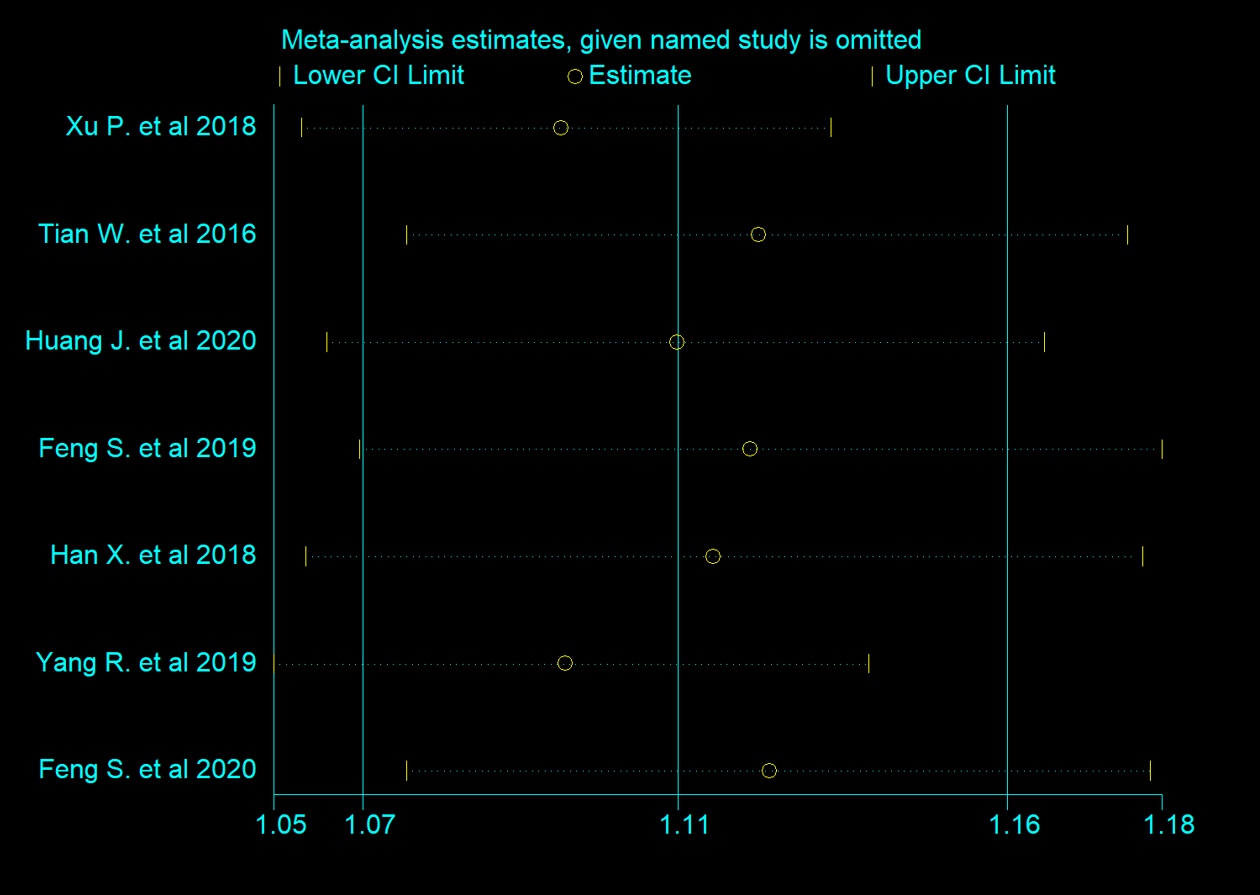


Fig. S1: Influence analysis results within the TiRobot group.


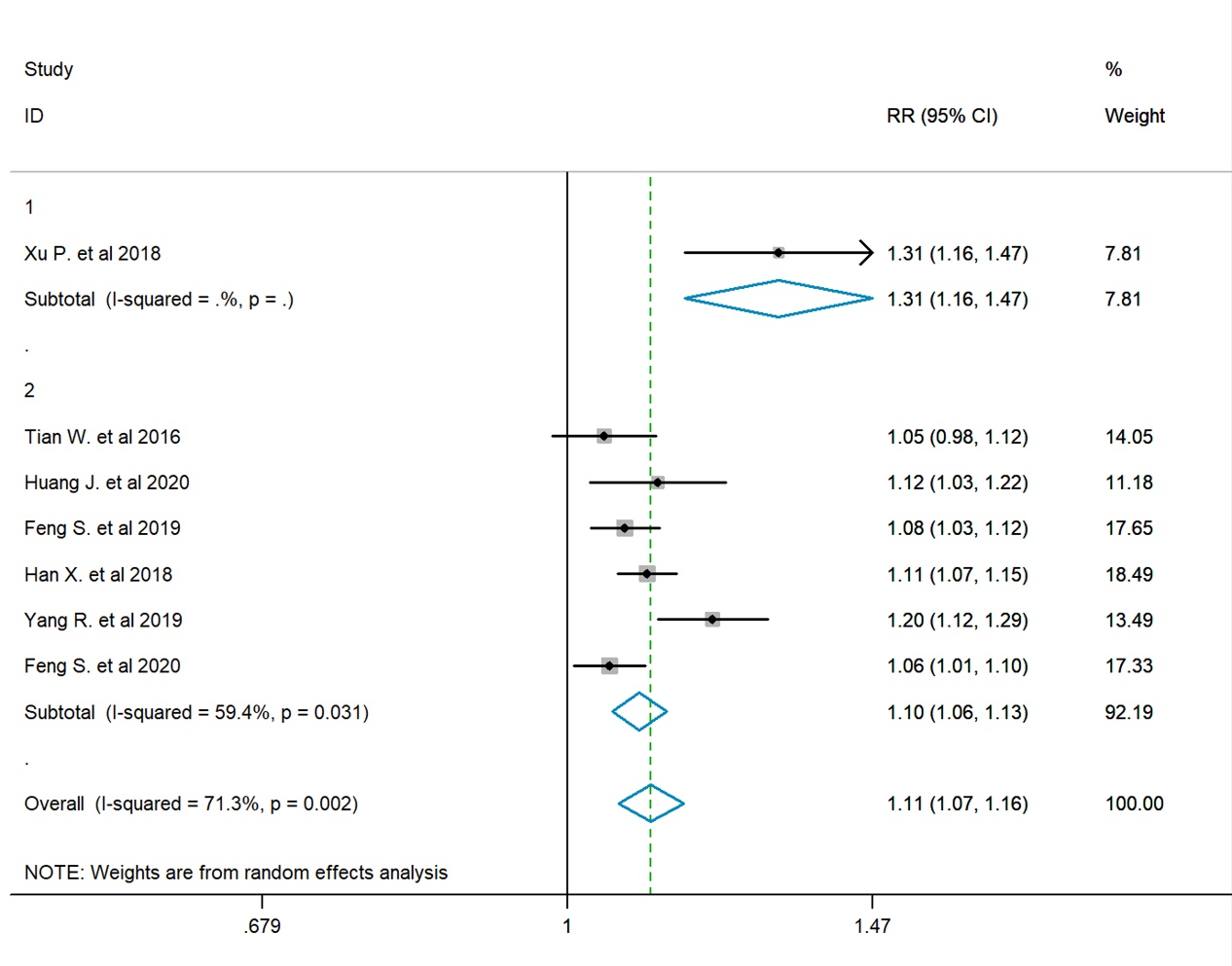


Fig. S2: Forest plot showing the separated results of the TiRobot group.


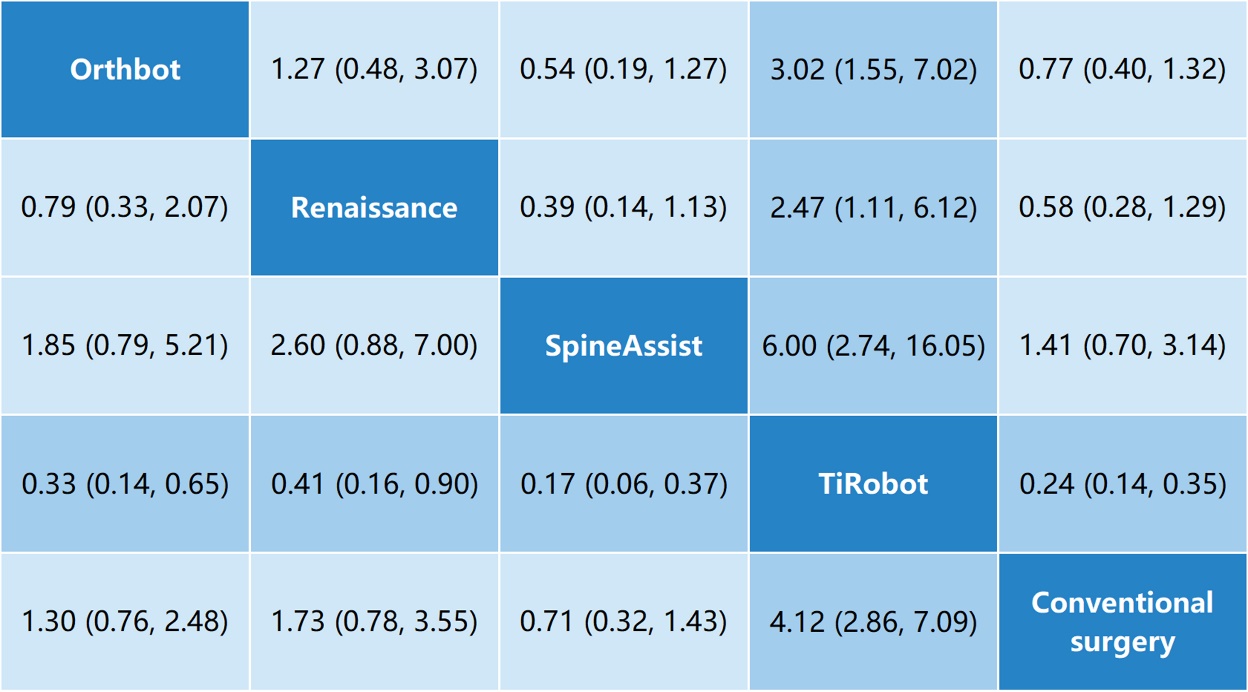


Fig. S3: Multiple comparisons for pedicle screw inserting accuracy based on the network consistency model (after exclusion).


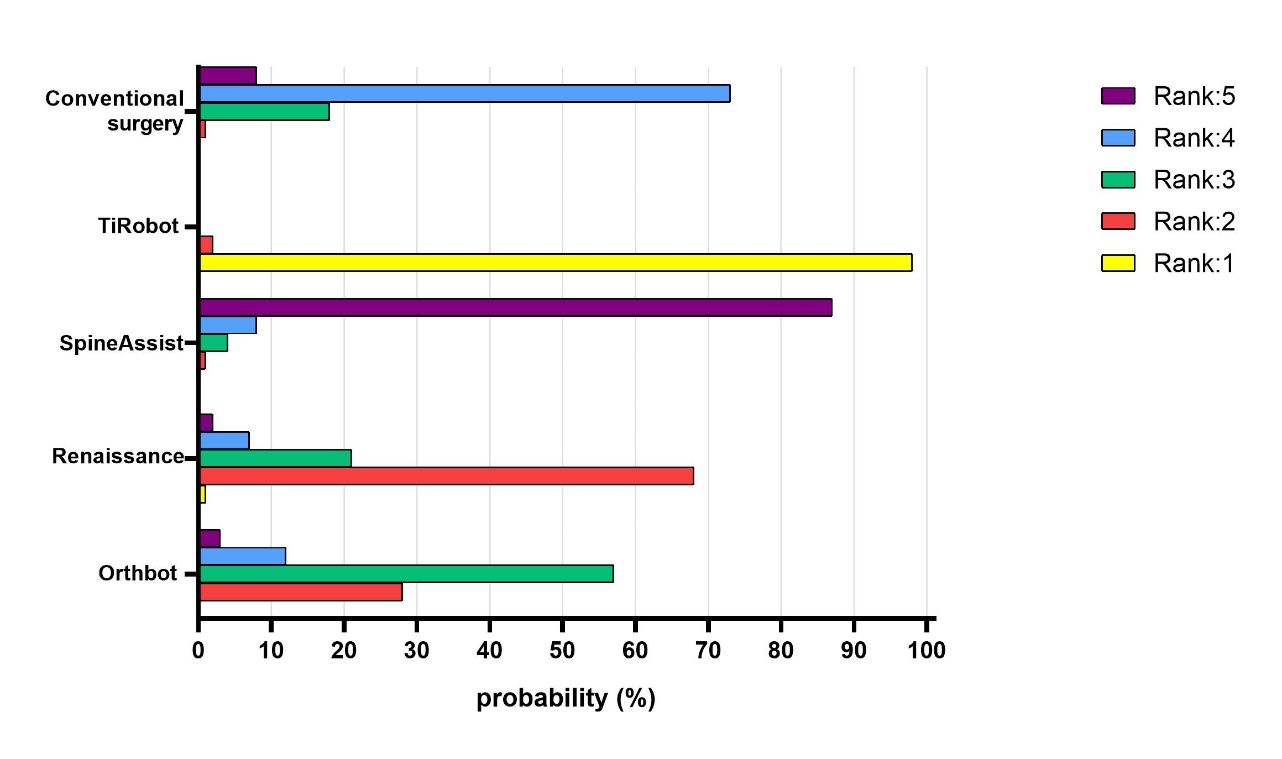


Fig. S4: Histogram showing the distribution of ranking probabilities of each operative technique in accuracy (after exclusion).
